# Quantitative Ultrasound Grayscale Analysis and Size of Benign and Malignant Solid Thyroid Nodules

**DOI:** 10.3390/tomography11120133

**Published:** 2025-11-27

**Authors:** Salahaden R. Sultan, Faisal Albin Hajji, Abdulrahman Alhazmi, Shahad Alamri, Abrar Alsulami, Ahmed Albukhari, Asseel Filimban, Bander Almutairi, Ahmad Albngali, Reham Kaifi, Mohammad Khayat, Mohammed Alkharaiji, Mohammad Khalil, Abrar Alfatni

**Affiliations:** 1 Department of Radiologic Sciences, Faculty of Applied Medical Sciences, King Abdulaziz University, Jeddah 21589, Saudi Arabia; 2 Department of Radiology, King Abdulaziz University Hospital, King Abdulaziz University, Jeddah 21589, Saudi Arabia; 3 College of Applied Medical Sciences, King Saud bin Abdulaziz University for Health Sciences, Jeddah 22384, Saudi Arabia; 4 King Abdullah International Medical Research Center, Jeddah 22384, Saudi Arabia; 5 Medical Imaging Department, Ministry of the National Guard—Health Affairs, Jeddah 21577, Saudi Arabia; 6 Department of Radiology, King Fahad General Hospital, Ministry of Health, Al-Madinah 42351, Saudi Arabia; 7 Department of Public Health, College of Health Sciences, Saudi Electronic University, Riyadh 11673, Saudi Arabia; 8 Department of Radiology, Faculty of Medicine, King Abdulaziz University, Jeddah 21589, Saudi Arabia

**Keywords:** ultrasound imaging, thyroid nodule, benign, malignant, quantitative analysis

## Abstract

Background: Ultrasound is the primary imaging modality for evaluating thyroid nodules, with echogenicity and nodule size serving as parameters for malignancy risk stratification. Though the TI-RADS classification system is standardized, interpretation varies among observers due to subjectivity, and can affect diagnostic consistency. This study aimed to evaluate the diagnostic and interobserver agreement of quantitative ultrasound gray-scale analysis and nodule area in differentiating benign from malignant solid thyroid nodules. Methods: This retrospective study reviewed 600 patients who underwent thyroid ultrasound at King Abdulaziz University Hospital, Jeddah, Saudi Arabia, in 2023 and 2024. Of these 600, 107 adult patients with 116 solid thyroid nodules (96 benign and 20 malignant) who subsequently underwent ultrasound-guided fine-needle aspiration were included in the final analysis. From B-mode ultrasound images, the grayscale median (GSM) values of each nodule and adjacent normal thyroid tissue were measured using Adobe Photoshop. The GSM ratio (GSMr) was calculated by dividing nodule GSM by normal tissue GSM. Nodule size, taken as cross-sectional area, was assessed using ImageJ software version 1.53. The Mann–Whitney *U* test was used to compare GSMr and the area between benign and malignant nodules. Inter-observer agreement was evaluated using the intraclass correlation coefficient (ICC). Results: Malignant nodules had significantly lower GSMr compared to benign nodules (malignant: median 0.76, IQR 0.27; benign: median 0.88, IQR 0.55, *p* = 0.02). Malignant nodules were also significantly larger than benign nodules (malignant: median 2.77 cm^2^, IQR: 5.08; benign: median 1.78 cm^2^, IQR 1.65, *p* = 0.02). Inter-observer reproducibility was excellent for both GSMr (ICC = 0.998) and area (ICC = 0.997). Conclusions: Quantitative ultrasound assessment of grayscale echogenicity and nodule area provides valuable diagnostic information for differentiating benign from malignant solid thyroid nodules. These objective measures may enhance diagnostic confidence and support more precise clinical decision-making in thyroid nodule evaluation.

## Simple Summary

Thyroid nodules are very common, and ultrasound is used to check whether they are likely to be cancerous or non-cancerous. Many ultrasound features are assessed visually, which can lead to differences between clinicians. In this study, we used computer-based measurements of how bright the nodules looked and how large they were, providing more objective information. Cancerous nodules tended to appear darker and larger, and these measurements were very consistent between observers. Our findings suggest that adding quantitative tools to routine thyroid ultrasound may improve diagnostic confidence of thyroid nodules.

## 1. Introduction

Thyroid nodules are discrete lesions within the thyroid parenchyma that differ radiologically from the surrounding tissue [1]. They represent one of the most frequently encountered abnormalities in endocrine imaging and are becoming increasingly prevalent in clinical practice [2]. Epidemiological data indicate that thyroid nodules occur in approximately 68% of the general population, with a malignancy rate ranging from 7% to 15% [3,4]. A recent review reported that the major clinical challenge in managing thyroid nodules lies in reliably distinguishing benign from malignant lesions using a standardized, cost-effective approach [5].

Ultrasound is the imaging modality of choice for evaluating and characterizing thyroid nodules owing to its wide availability, noninvasive nature, absence of ionizing radiation, and cost-effectiveness [6]. Ultrasound classification systems—such as the American College of Radiology Thyroid Imaging Reporting and Data System (ACR TI-RADS), and those of the Korean Society of Thyroid Radiology (K-TIRADS) and the European Thyroid Association (EU-TIRADS)—underpin the ultrasound-based assessment and management of thyroid nodules [7]. These systems provide structured frameworks for risk stratification, allowing for a standardized interpretation of sonographic features and guiding clinical decision-making regarding ultrasound-guided fine-needle aspiration (FNA), helping identify which nodules warrant biopsy, which require short-term ultrasound surveillance, and which can safely be excluded from further evaluation [8]. ACR TI-RADS classifies nodules based on their composition, echogenicity, shape, margins, and echogenic foci, with increasing scores corresponding to higher malignancy risk [5,9]. Each feature is scored—from 0 for benign characteristics such as cystic or spongiform composition to 3 points for highly suspicious findings such as very hypoechoic echogenicity, taller-than-wide shape, extrathyroidal extension, or punctate echogenic foci [9,10]. The total score categorizes nodules from TR1 (benign) to TR5 (high suspicion), with size thresholds determining whether FNA or ultrasound follow-up is required; TR1–TR2 require no FNA, whereas TR3–TR5 have graduated recommendations based on size, beginning at ≥1.5 cm for TR3 and ≥1.0 cm for TR5, alongside defined follow-up intervals [9,10]. Meaningful interval growth is defined as >20% in two dimensions with >2 mm increase, or >50% volume increase, prompting re-evaluation [9,10]. K-TIRADS categorizes nodules from K-TIRADS 1 (no nodule) to K-TIRADS 5 (high suspicion) based on echogenicity and three suspicious features—punctate echogenic foci, non-parallel orientation, and irregular margins—with malignancy risks ranging from <3% in benign spongiform or cystic nodules (K-TIRADS 2) to >60% in high-suspicion nodules (K-TIRADS 5), and biopsy recommended for nodules > 1–2 cm depending on category [11,12,13]. Similarly, EU-TIRADS classifies nodules from EU-TIRADS 1 (no nodule) to EU-TIRADS 5 (high suspicion), with benign cystic/microcystic nodules designated EU-TIRADS 2, and higher-risk nodules identified by features such as non-oval shape, irregular margins, microcalcifications, or marked hypoechogenicity [14]. FNA thresholds are >20 mm for EU-TIRADS 3, >15 mm for EU-TIRADS 4, and >10 mm for EU-TIRADS 5, with optional biopsy or follow-up for smaller high-risk nodules [14].

The echogenicity and size of thyroid nodules remain active areas of investigation. Echogenicity denotes the grayscale brightness of a nodule relative to surrounding thyroid parenchyma and adjacent strap muscles and is commonly categorized as hyperechoic, isoechoic, or hypoechoic. This qualitative stratification underpins major reporting systems and is integral to standardized malignancy risk assessment [15]. Hypoechogenic nodules increase malignancy likelihood, whereas iso- and hyperechoic nodules are more likely to be benign [9,16]. The relationship between thyroid nodule size and malignancy risk remains controversial, and it remains unclear whether thyroid nodule size predicts the risk of malignancy as the current evidence is conflicting [17]. A meta-analysis suggested that nodules between 3 and 5.9 cm have a higher malignancy risk (26%) than smaller ones, while nodules ≥ 6 cm have a lower risk (16%) [18]. However, smaller nodules (<2 cm) have also be shown to be associated with malignancies [19]. These associations highlight the complexity of size as a predictive parameter, underscoring the need for a more nuanced approach to thyroid nodule risk stratification.

Ultrasound assessment of thyroid nodules remains operator-dependent: significant observer variability affects diagnostic accuracy and reproducibility. Studies have demonstrated only moderate operator agreement when classifying nodule echogenicity [20,21]. The inconsistency stems from the qualitative, subjective nature of echogenicity evaluation, which depends on visually comparing nodule brightness with surrounding thyroid parenchyma and adjacent muscles, as well as operator experience. Furthermore, a systematic review and meta-analysis evaluating more than 7000 thyroid nodules reported that the diagnostic performance of ultrasound risk stratification systems in indeterminate thyroid nodules was, based on cytology, moderately successful in identifying malignancy [22]. These results suggest the need to incorporate quantitative ultrasound parameters to improve diagnostic accuracy and promote clinical consistency. Given the inconsistency associated with subjective ultrasound assessment of thyroid nodule echogenicity, and due to the limitations of quantitative studies evaluating nodule echogenicity and nodule area, this study aimed to assess the diagnostic performance and interobserver agreement of computer-assisted grayscale median ratio (GSMr) analysis and nodule area measurement in differentiating benign from malignant solid thyroid nodules.

## 2. Methods

### 2.1. Study Design and Population

This retrospective study was conducted using data from the radiology archives of King Abdulaziz University Hospital in Jeddah, Saudi Arabia. The dataset included patients who underwent thyroid ultrasound examinations in 2023 and 2024, with data collected from January to April 2025. A total of 600 patient records were reviewed. All data were de-identified, and ethical approval was obtained from the institutional review boards (reference no. 33-25). Inclusion criteria were adult patients with predominantly solid thyroid nodules assessed by ultrasound and definitively diagnosed as benign or malignant through cytological evaluation following ultrasound-guided fine-needle aspiration, with no prior thyroid surgery. Patients were excluded if they had non-solid nodules, or no thyroid nodules on ultrasound, no ultrasound images or cytology reports, or had a history of thyroid surgery.

### 2.2. Data Acquisition and Ultrasound Image Analysis

**Figure 1:**
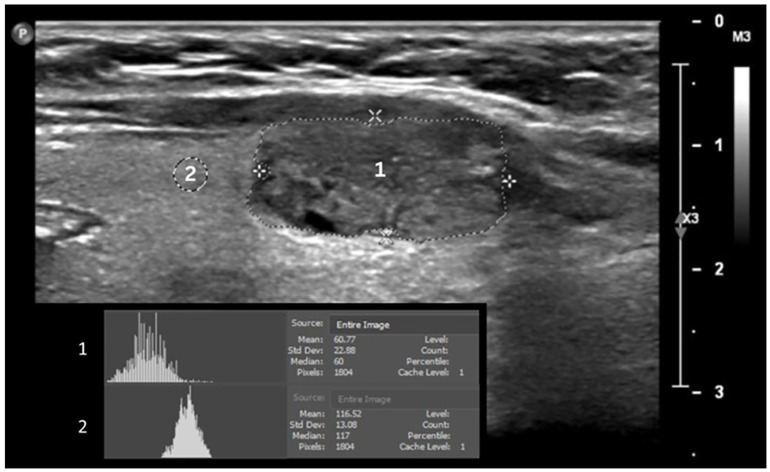
Measurement of echogenicity. Gray-scale median of thyroid nodules (1) and normal thyroid tissues (2).

**Figure 2:**
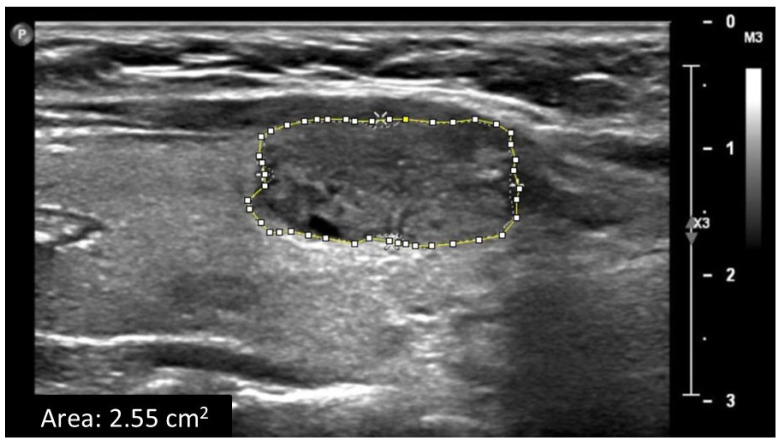
Measurement of thyroid nodule area.

Patient demographics and clinical data, including age, gender, and chronic diseases (i.e., hypertension and diabetes), were extracted from the patient clinical record. Thyroid ultrasound examinations were performed using a linear-array transducer of the Philips ultrasound imaging system. The thyroid was assessed bilaterally. Two-dimensional B-mode images were acquired. Ultrasound B-mode settings were optimized for high-quality grayscale images, including depth, focus point, and the gain. From each B-mode ultrasound image, grayscale median (GSM) values of the entire solid thyroid nodules and from adjacent normal thyroid tissue were obtained using Adobe Photoshop software and used as measurements of echogenicity (Figure 1). The GSM ratio (GSMr) was calculated using the following equation: GSMr = solid nodule GSM ÷ normal thyroid tissue GSM. The area of the solid thyroid nodule was measured off-line using ImageJ software (Figure 2). Grayscale histogram analysis is a well-established method for quantitatively assessing echogenicity in ultrasound imaging, providing an objective measure of pixel intensity within a defined region of interest. Its validity has been demonstrated through direct comparisons using other imaging software, such as ImageJ, showing comparable reliability for grayscale quantification [23]. Adobe Photoshop is widely used for this purpose and has been applied in various clinical settings, including quantitative ultrasound analysis of subcutaneous tissues [24]. Furthermore, histogram-based analysis has been shown to improve the reproducibility of carotid plaque echogenicity measurements and to reduce the subjectivity inherent in visual interpretation [25]. The presence of predominantly solid thyroid nodules on all ultrasound images was confirmed independently by a consultant sonographer and a consultant radiologist, each with more than seven years of clinical experience. GSM and nodule area were then independently assessed by two senior sonographers, both of whom routinely perform thyroid ultrasound examinations and have more than five years of clinical experience. The observers were blinded to each other’s assessments, to all clinical information, to the results of other imaging examinations, and to the cytology outcomes, ensuring unbiased evaluation. All cytology interpretations were conducted by a consultant pathologist.

### 2.3. Statistical Analysis

The average GSMr and area values from both observers were analyzed to differentiate between benign and malignant solid thyroid nodules. The Mann–Whitney *U* test was applied to evaluate differences in GSMr and area between the two groups. Inter-observer reproducibility for GSMr and area was examined using the intraclass correlation coefficient (ICC), and Bland–Altman analysis was used to determine measurement bias and limits of agreement. All statistical analyses were performed in a fully blinded manner by a public health and epidemiology statistician with more than five years of experience. The significance threshold was set at 0.05. Analysis were performed using IBM SPSS Statistics version 21 (Armonk, NY, USA: IBM Corp.) and GraphPad PRISM 7 (La Jolla, CA, USA).

## 3. Results

### 3.1. Patient Characteristics

Ultrasound B-mode images of 116 solid thyroid nodules (96 benign and 20 malignant) from 107 patients with solid thyroid nodules were evaluated. Of the 107 patients included in this study, 94 were female (79 benign and 15 malignant), 22 were male (8 benign and 5 malignant), 19 had hypertension (17 benign and 2 malignant), and 24 were diabetic (22 benign and 2 malignant). The average GSM of the nodules was 84.90 ± 28.43, for the benign nodules, GSM = 90.70 ± 23.86 and, for the malignant nodules, GSM = 77.52 ± 25.37. The average GSM of adjacent normal thyroid tissue was 92.61 ± 25.11, with values of 90.70 ± 23.86 in benign cases and 101.77 ± 29.00 in malignant cases. The mean nodule area was 2.66 ± 2.2 cm^2^, with benign nodules averaging 2.03 ± 1.15 cm^2^ and malignant nodules 3.62 ± 2.47 cm^2^. A summary of patient information and features of thyroid nodules are presented in Table 1.

### 3.2. GSMr and Area of Benign and Malignant Solid Thyroid Nodules

**Figure 3:**
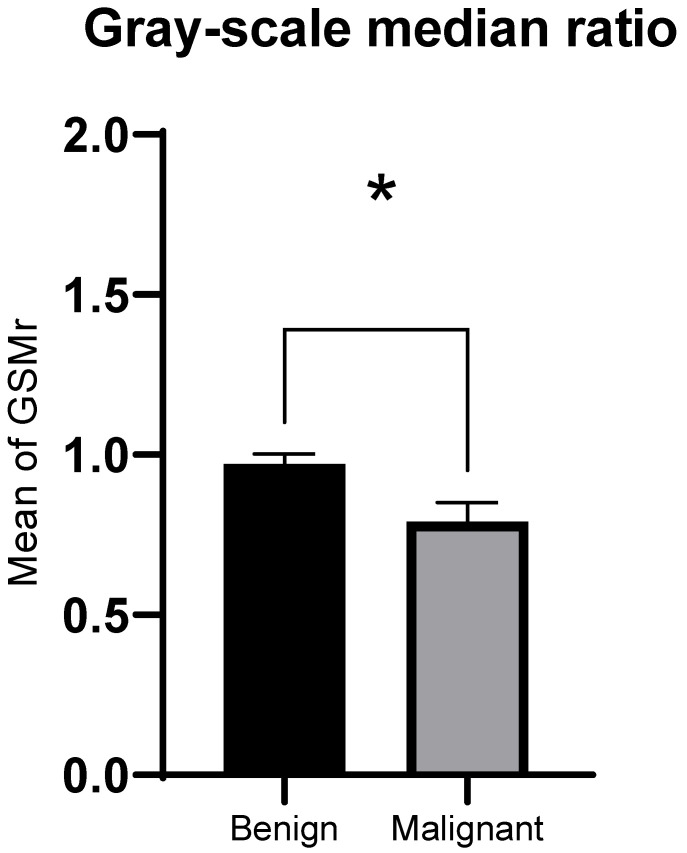
Gray-scale median ratio (GSMr) (mean ± SEM). * *p* ≤ 0.05 using Mann–Whitney U test between malignant and benign thyroid nodules.

**Figure 4:**
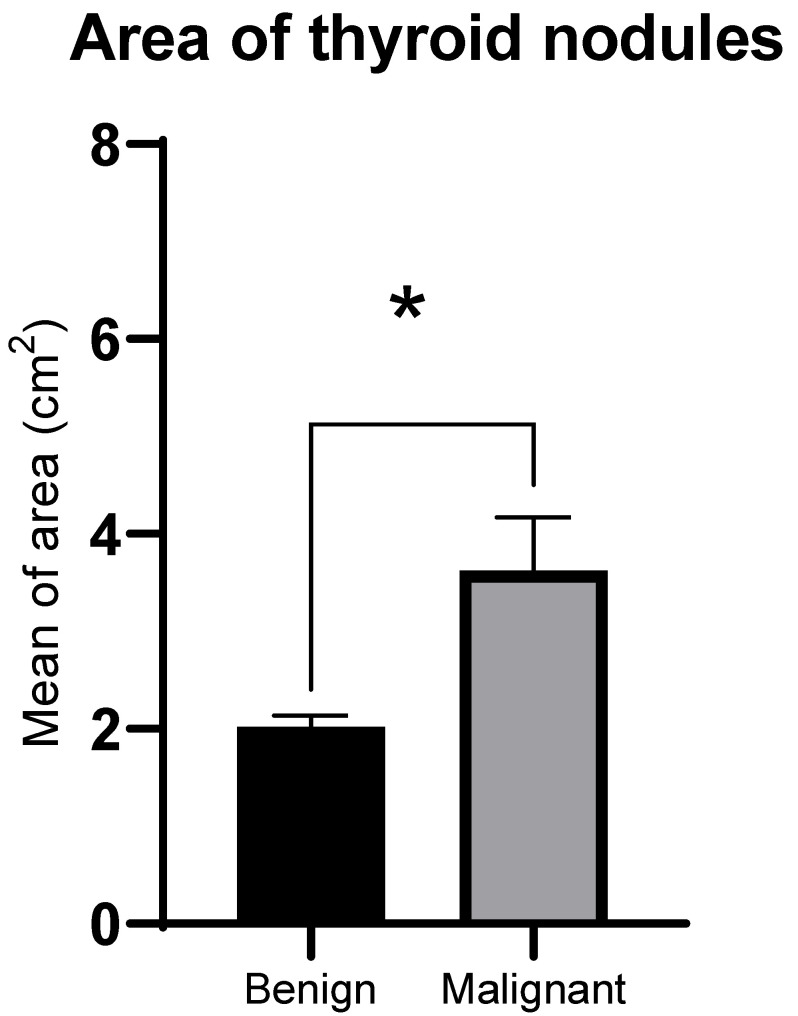
Area of thyroid nodules (mean ± SEM). * *p* ≤ 0.05 using Mann–Whitney U test between malignant and benign thyroid nodules.

The GSMr of malignant solid thyroid nodules was significantly lower compared to benign nodules (median 0.76, interquartile range (IQR) 0.27 for malignant nodules; median 0.88, IQR 0.55 for benign nodules; Z = −2.28, *p* = 0.02, Figure 3). Areas of malignant solid thyroid nodules were significantly larger compared to benign nodules (median 2.77 cm^2^, IQR 5.08 cm^2^ for malignant nodules; median 1.78 cm^2^, IQR 1.65 cm^2^ for benign nodules; Z = −2.01, *p* = 0.02, Figure 4).

### 3.3. Inter-Observer Agreement of GSMr and Area

**Figure 5:**
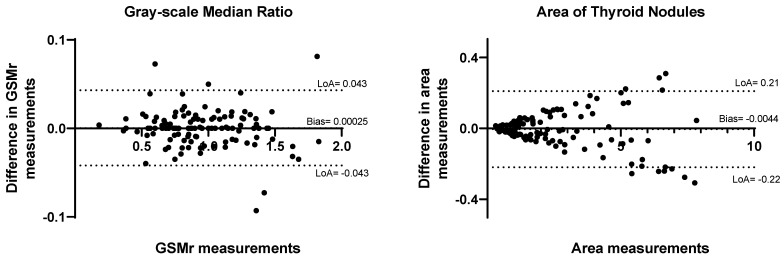
Inter-observer reproducibility of gray-scale median ratio (GSMr) and area. Bland–Altman agreement of GSMr and area. LOA = limit of agreement.

The inter-observer agreement of GSMr and median values was excellent, with ICC values of 0.998 and 0.997, respectively. The bias in GSMr measurements between observers was −0.00025 ± 0.022 (limits of agreement [LoA]: −0.043–0.043, Figure 5). Bias in area measurements between observers was −0.0044 ± 0.11 cm^2^ (LoA: −0.22–0.21, Figure 5).

## 4. Discussion

This study assessed quantitative computer-assisted GSMr analysis with nodule area measurement to provide a more objective and reproducible method for distinguishing benign from malignant solid thyroid nodules, offering a promising approach for enhancing thyroid nodule evaluation. The study findings demonstrated that malignant nodules exhibited significantly lower GSMr than benign nodules, indicating reduced echogenicity relative to adjacent normal thyroid tissue. Furthermore, malignant nodules demonstrated significantly larger areas than benign nodules, suggesting that quantitative size assessment may complement echogenicity evaluation in malignancy risk stratification. The study showed excellent inter-observer reproducibility for both GSMr and area measurements, addressing a critical limitation of conventional qualitative ultrasound assessment where substantial observer variability may affect diagnostic consistency. These findings support the integration of objective, quantitative ultrasound parameters into clinical practice as an additional tool to existing risk stratification systems, potentially enhancing diagnostic confidence.

A recent review of ultrasound features, with their corresponding cytopathology findings for the most common thyroid entities encountered in routine practice, reported that benign nodules typically appear small, predominantly cystic, isoechoic or hyperechoic, or spongiform, corresponding to cytology showing macrofollicles in abundant colloid with low cellularity and macrophages in cystic areas [26]. Malignant and suspicious nodules, by contrast, are usually solid, markedly hypoechoic, irregular, taller than wide, or contain microcalcifications, correlating with hypercellular FNAs and minimal colloid [26,27]. Follicular-patterned lesions show nonspecific solid iso- to hypoechoic ultrasound features and microfollicular hypercellularity on cytology but cannot be distinguished as adenoma versus carcinoma cytologically [26]. Papillary carcinoma typically shows hypoechogenicity, microcalcifications, and irregular margins, matching cytology with classic nuclear grooves, pseudoinclusions, and psammoma bodies [26,27]. Medullary carcinoma may present as solid hypoechoic or multifocal nodules with nodal metastases and cytology demonstrating spindled or plasmacytoid cells with possible amyloid, whereas anaplastic carcinoma appears as a large heterogeneous mass with extrathyroidal extension and cytology showing highly pleomorphic malignant cells and necrosis [26,28]. Across these entities, hypoechogenicity remains a strong ultrasound predictor of malignancy, attributable to the densely packed follicular cells and reduced colloid content characteristic of malignant nodules [16,29].

The current study’s finding—that malignant nodules demonstrated lower GSMr values—corroborates evidence that hypoechogenicity correlates with progressively higher cancer risk [9,15,30,31]. It has been demonstrated that markedly and moderately hypoechoic nodules exhibited significantly higher malignancy rates compared to mildly hypoechoic lesions, with the ACR TI-RADS system assigning two points to marked hypoechogenicity in recognition of its substantial contribution to malignancy risk assessment [9,10]. However, the qualitative nature of conventional echogenicity assessment, which relies on subjective visual comparison of nodule brightness relative to surrounding thyroid parenchyma and adjacent muscles, makes it particularly susceptible to inter-observer variability: recent studies report concern for discrimination between mildly/moderately and markedly hypoechoic nodules [21]. The quantitative GSM-based approach employed in the present study addresses this limitation by providing objective numerical values that eliminate subjective interpretation. Wu et al. (2016) similarly demonstrated that quantitative echoic indexes, calculated as grayscale pixel intensity ratios between nodule, thyroid parenchyma, and strap muscle regions, could effectively discriminate malignant from benign nodules while providing reproducible measurements independent of observer bias [32]. The excellent inter-observer agreement achieved in our study supports the clinical utility of quantitative grayscale analysis as an adjunct to conventional ultrasound evaluation [20,32].

Clinical practices often consider thyroid nodule size, favoring surgical management of larger nodules primarily due to compressive symptoms rather than their association with malignancy risk [33,34], and current evidence-based guidelines emphasize that FNA decisions should be driven primarily by ultrasound risk categories, with size thresholds serving only as secondary modifiers within each risk category: the 2023 European Thyroid Association guidelines, for example, recommends that fine-needle aspiration decisions be based primarily on nodule risk category rather than size alone, though size thresholds are applied within specific risk categories [16]. A recent systematic review investigated the relationship between thyroid nodule size and malignancy and examined the association between FNA results and malignancy rates in small and large nodules, reporting that it is difficult to draw any definitive conclusions regarding the association between nodule size and malignancy due to heterogeneity in size–malignancy associations across available evidence [35]. For example, a Cavello et al. (2017) stated that size was inversely related to cancer risk [36]. Others conversely concluded that thyroid nodule size was not associated with malignancy risk in nodules ≥ 4 cm and patients with thyroid nodules ≥ 4 cm and benign cytology should not be routinely referred for thyroidectomy [37]. Other studies reported that high rates of malignancy and false negatives among thyroid nodules over 3 cm indicated that thyroidectomy should be considered for such individuals even if the cytology is benign [38], and that malignancy rate was higher for nodules over 3 cm [39]. Still, other studies have found that the risk of follicular carcinomas and other rare thyroid malignancies increases as nodules enlarge [40,41]. An inverse relationship has been reported: smaller nodules show higher malignancy rates due to preserved suspicious features, whereas larger nodules often undergo degenerative changes that obscure malignant characteristics [42]. It is worth noting that the current method considered for measuring nodule size considers both length and width. It has been reported that shape and dimensional ratios, including the taller-than-wide morphology, have been considered useful as indicators of malignancy [43,44], supporting the use of area measurement as an additional predictor of thyroid nodule characteristics and their potential malignancy risk, as applied in the present study. The observed association between larger nodule area and malignancy may reflect the cumulative effect of sustained proliferative activity driven by underlying molecular alterations in malignant thyroid cells, resulting in progressive nodule enlargement prior to clinical detection. Malignant nodules frequently exhibit dysregulation of key signaling pathways, including MAPK and PI3K/AKT, and harbor driver mutations such as BRAFV600E, which collectively promote accelerated cellular proliferation and reduced colloid content [45,46]. Nodules with high-risk molecular profiles have been shown to grow significantly faster than benign lesions [47,48], and their densely packed cellular architecture may further facilitate expansion [49]. This proliferative behavior is supported by a permissive tumor microenvironment enriched with angiogenic mediators, immune interactions, and cancer-associated fibroblasts that enhance malignant cell survival and growth [46,50]. However, the size–malignancy relationship remains complex, as detection bias may influence observed patterns in which smaller malignant nodules may go unrecognized or unbiopsied until they enlarge and become symptomatic or palpable.

Advanced technologies in ultrasound imaging, including elastography, contrast-enhanced ultrasound (CEUS), and three-dimensional ultrasound (3DUS), have emerged as adjuncts to conventional B-mode ultrasound for improving thyroid nodule characterization and diagnostic confidence. These imaging technologies could be particularly valuable in the evaluation of indeterminate nodules, where standard grayscale imaging alone does not provide sufficient information for accurate risk stratification [51]. Ultrasound elastography assesses tissue stiffness based on the principle that malignant thyroid nodules are generally stiffer due to increased cellular density, fibrosis, and desmoplastic reaction [52,53], with a meta-analysis reporting satisfactory overall accuracy of elastography for distinguishing benign from malignant indeterminate thyroid nodules [54]. CEUS enhances thyroid nodule evaluation by visualizing macro- and microvascularization after administering microbubble contrast agents. Malignant nodules are typically characterized by hypo-enhancement, heterogeneous enhancement, irregular peripheral enhancement combined with abnormal internal enhancement patterns, and a slow wash-in and wash-out curve relative to normal thyroid parenchyma. In contrast, benign nodules usually demonstrate homogeneous and intense enhancement, smooth rim enhancement, and a fast-in and slow-out perfusion pattern [55]. 3DUS provides a volumetric assessment of thyroid nodules, enabling comprehensive visualization of nodule margins, shape, and spatial relationships with surrounding structures [56]. This technique reduces operator dependency, thereby improving interobserver reproducibility compared to 2D imaging [57], comparable to computed tomography for volume estimation [58]. These advanced ultrasound imaging technologies have also been reported to improve procedural guidance, improving the yield of FNA and helping identify suspicious regions within a nodule for targeted sampling, thus minimizing sampling errors, and enhancing diagnostic confidence and enabling accurate intervention procedures [59,60]. These advantages suggest that integrating multiparametric ultrasound offers a promising approach to achieving more accurate thyroid nodule risk stratification than relying on single imaging techniques alone. However, further investigation and standardization of these technologies are still required to enhance the diagnostic accuracy and reliability of ultrasound imaging in thyroid cancer detection.

Artificial intelligence (AI) has emerged as a rapidly evolving component of thyroid nodule evaluation, offering new capabilities that complement traditional ultrasound assessment [5]. Radiomics enables automated extraction of quantitative imaging features that characterize tissue heterogeneity, morphology, and texture, ready for subsequent analysis using machine learning or deep learning systems to support diagnostic and predictive modeling in precision medicine [61]. Radiomics outputs can be visualized directly by clinicians when integrated into computer-aided diagnosis platforms, improving accuracy and reducing interobserver variability in thyroid nodule assessment [62]. Despite rapid growth and expanding commercial interest, there is a need for methodological standardization and clinical validation before widespread implementation [61]. AI advancements are expected to enhance workflow efficiency and diagnostic precision for radiologists and ultrasound specialists [61,62], yet expert clinical judgment remains essential for integrating imaging with patient history, interpreting atypical cases, and addressing ethical, legal, and interpersonal aspects of care [5].

This study has several limitations that should be considered. First, this study was conducted as a retrospective cross-sectional analysis using ultrasound examinations and their corresponding cytology results, without any longitudinal clinical follow-up period. The analysis was restricted to solid thyroid nodules, excluding cystic and mixed lesions, which limits generalizability to the broader spectrum of thyroid nodule types encountered in routine clinical practice. As this study represents an initial exploration of GSMr and nodule area, anechoic or predominantly cystic nodules were excluded because of their inherently low echogenicity, which may not be suitable for GSMr-based evaluation and could confound interpretation of the low GSMr values observed in malignant solid nodules. Although Adobe Photoshop has been applied in several clinical applications and shown to provide reliable and reproducible gray-scale measurements, further validation of its use specifically for quantifying thyroid nodule echogenicity is warranted. Future studies comparing Photoshop-based measurements with dedicated ultrasound analysis software may help standardize quantitative assessment of thyroid nodules. GSM and area measurements were performed offline, allowing observers to analyze images without time constraints, which likely improved precision and reproducibility. This differs from real-world clinical settings, where clinicians must complete examinations quickly to manage high patient volumes, potentially reducing measurement accuracy and consistency. As this study was designed as a retrospective exploratory analysis, no a priori sample size calculation was performed. The current sample size, particularly the number of malignant nodules, reflects the number of available cases that met inclusion criteria during the study period. However, the study does provide preliminary observations regarding the potential diagnostic role of quantitative GSMr and nodule area. Future large prospective studies with power-based sample size calculations that include the full spectrum of thyroid nodules with standardized TI-RADS documentation are needed to validate the present study results and to further assess diagnostic indicators such as sensitivity, specificity, and optimal GSMr cut-off values.

## 5. Conclusions

This pilot retrospective study demonstrates that malignant thyroid nodules exhibit significantly lower GSMr values and larger nodule areas than benign nodules, with excellent inter-observer reproducibility for both measurements. While these preliminary findings suggest that quantitative ultrasound may offer a more standardized and objective approach to reduce variability in thyroid nodule evaluation, the limited number of malignant cases warrants cautious interpretation. Large prospective studies are required to determine the clinical utility of GSMr and area thyroid nodule evaluation.

## Figures and Tables

**Table 1:** Patient information and features of thyroid nodules.

Variable	Patient (*n* = 107)	Benign (*n* = 96)	Malignant (*n* = 20)
Gender			
Female, *n* (%)	94 (87.9)	79	15
Male, *n* (%)	13 (12.1)	8	5
Hypertension, *n* (%)	19 (17.8)	17	2
Diabetes Mellitus, *n* (%)	24 (22.4)	22	2
Nodules evaluated, *n*	116	96	20
Nodule GSM, median	84.5	86.5	80
Thyroid tissue GSM, median	90	86.5	101.5
Nodule area (median, cm^2^)	1.8	1.7	2.7

## Data Availability

The data presented in this study are available on request from the corresponding author due to ethical restrictions.
